# Red List of Liverworts and Hornworts of Sicily

**DOI:** 10.3390/plants15030398

**Published:** 2026-01-28

**Authors:** Mattia Letizia Marino, Maria Giovanna Dia, Marta Puglisi, Patrizia Campisi

**Affiliations:** 1 Department of Agricultural, Food and Forest Sciences, University of Palermo, Viale Delle Scienze, Bldg. 5, I-90128 Palermo, Italy; mattialetizia.marino@unipa.it; 2 Independent Researcher, Via Monte Bonifato, 191, I-91011 Trapani, Italy; m.giovanna.dia@people.unipa.it; 3 Department of Biological, Geological and Environmental Sciences, University of Catania, Via A. Longo 19, I-95125 Catania, Italy; 4 Department of Biological, Chemical and Pharmaceutical Sciences and Technologies, University of Palermo, Via Archirafi 38, I-90123 Palermo, Italy; patrizia.campisi@unipa.it; 5 National Biodiversity Future Center (NBFC), University of Palermo, Piazza Marina 61 (c/o Palazzo Steri), I-90133 Palermo, Italy

**Keywords:** threatened species, extinction risk, Sicilian flora conservation, IUCN categories, IUCN criteria

## Abstract

This study provides an updated conservation status of all liverworts and hornworts in Sicily, evaluated according to the IUCN Guidelines for Application of IUCN Red List Criteria at the Regional Level. Of the assessed taxa, 40 taxa (31.74%) are assigned to a risk category and, specifically, 18 (14.29%) are classified as Critically Endangered (CR), 13 (10.32%) as Endangered (EN), and 9 (7.14%) as Vulnerable (VU). In addition, 13 taxa (10.32%) are classified as Near Threatened, and 20 (15.87%) as Data Deficient; however, many of these are likely to qualify for inclusion in one of the IUCN Red List threat categories following further field surveys and data acquisition. The remaining 52 taxa (41.27%) are classified as Least Concern (LC).

## 1. Introduction

Overall, Sicily hosts a medium–high bryophyte species richness compared to other Italian regions [1], with a total of 613 taxa known to date, including 487 mosses and 126 liverworts and hornworts.

Investigations of the bryophyte flora began in the early nineteenth century and have progressively led to a fairly comprehensive exploration of the island, despite intermittent periods of reduced research activity. As a result, a substantial body of knowledge has been accumulated on the bryophyte species occurring in the region, encompassing a wide range of environments, from mountainous areas to coastal zones, and from protected natural and archaeological sites to urban contexts, e.g., [2,3].

The surveys have highlighted the presence in Sicily of several interesting taxa due to peculiar taxonomic and phytogeographical aspects, often worthy of conservation, e.g., [4,5,6]. In consideration of their interest, a few of these taxa have been further studied to define their population size and trend, with the aim of providing useful information for their protection and monitoring [7].

In recent years, research on bryophytes has particularly focused on conservation issues. In fact, although the need to adopt measures to safeguard biodiversity in Sicily concerns all its components, it appears even more urgent for those taxonomic groups, such as bryophytes, which are very little known and neglected by most of the legislative instruments issued at the national and regional level to protect nature.

In addition to the lack of awareness and attention by the part of public opinion and administrators, we also observe the existence of various threats related to anthropogenic disturbance in many contexts of the island, which cause alteration and destruction of habitats, and lead to species rarefaction or even extinction. In this regard, it is sufficient to look at the data relating to the surface covered by fires in the last decade or those referring to land consumption to notice how the values are incredibly high, although fluctuating from one year to the next, placing Sicily among the most affected regions in Italy [8,9] and raising concerns about the conservation status of habitats and species.

Moreover, it should be remembered that the Sicilian territory has undergone profound transformations over the course of its long history, and the island is today among the most densely urbanized Italian regions. Uncontrolled construction in coastal areas and also in inland areas and sometimes even in semi-natural and natural areas, the canalization of watercourses and the continuous capture of springs, in addition to fires, constitute the main causes of risk and contribute to the start of a process of desertification, clearly also connected to the current climatic variations.

Nevertheless, many places of particular environmental interest still persist in Sicily, and, for their conservation, the Sicilian Regional Administration has already established four Parks and many Nature Reserves. However, regarding bryophytes, these protected areas are insufficient to protect the threatened species, since many interesting species occur in areas which are not at all protected, where they occupy particular niches.

As biodiversity conservation has become a crucial issue in a world increasingly affected by human impact, using tools to prevent further damage to species and ecosystems has become a priority.

In this regard, Red Lists play a significant role since protection policies largely stand on the information they provide. They constitute the prerequisite for the adoption of adequate conservation measures, as they are the scientific bases used to target rare and threatened species and to identify habitats in need of protection. Moreover, they contribute to raise community awareness on biodiversity issues.

As their value is widely recognized, in recent decades, Red Lists referring to distinct taxonomic groups have been drawn up all over the world at different scales, from global to the regional level.

With reference to bryophytes, recently, a comprehensive Red List of European Bryophytes [10] and several Red Lists in different countries of the continent have been carried out, e.g., [11,12,13,14,15,16,17,18].

In Italy, after the first Red List of bryophytes was published more than thirty years ago [19] and based on the first IUCN categories drawn up in 1978, some assessments of single species had been published [20,21,22,23,24,25,26] and the assessments of a group of bryophytes, most of all included in the Annexes II, IV and V of the “Habitat” Directive 92/43/EEC and the Berne Convention [27]. Only recently two complete and updated Red Lists have been published, one of liverworts and hornworts [28] and one of mosses [29], both of which follow the latest IUCN criteria and categories [30,31,32].

As for Sicilian bryophytes, a first attempt to give an overview of their conservation status was made in 2003 by Campisi, Aiello and Dia [33], who published a preliminary work to the drafting of the Red List in which the rarest and most threatened taxa were pointed out but without a formal assessment.

In this paper, we aim to establish a Red List which takes stock of the conservation status of all liverworts and hornworts of Sicily, providing an assessment based on the IUCN criteria [30,31] and the latest version of IUCN Guidelines [34].

Although significant knowledge gaps still remain, as data on population trends and numbers of individuals are largely missing and information on species distribution is also incomplete, we consider this Red List a necessary starting point for further studies and investigations that can lead to full knowledge of the risk status of Sicilian liverworts. After all, Red Lists are a dynamic evaluation tool and are therefore subject to periodic changes due to the increase in information over time, as in the case of the Red List of the bryophytes of Britain in which in the space of about a decade 69% of the species previously included in a threatened category have been transferred to a new category [16].

## 2. Results

Overall, 126 taxa were evaluated, including 4 hornworts (*Anthocerotophyta*) and 122 liverworts (*Marchantiophyta*), which are reported in Table 1.

**Figure 1:**
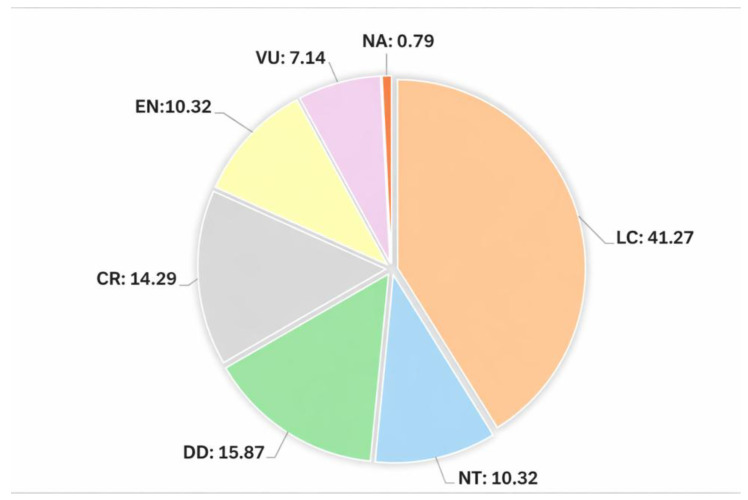
Percentage distribution of assessed species across IUCN Red List categories in Sicily.

The Red List of hornwort and liverwort species in Sicily comprises 41 threatened taxa (32.54%) (Table 1, Figure 1). In particular, 18 taxa (14.29%) are Critically Endangered (CR), 13 (10.32%) and 9 (7.14%) are Endangered (EN) and Vulnerable (VU), respectively. The other categories represented are Near Threatened (NT) (13 taxa, 10.32%), Data Deficient (DD) (18 taxa, 15.87%), Least Concern (LC) (52 taxa, 41.27%) and Not Applicable (NA) (1 taxon, 0.79%) assigned to a vagrant species, defined by IUCN [30] as “A taxon that is currently found only occasionally within the boundaries of a region”. We have not assessed any taxon as regionally Extinct (RE), because we have no evidence or reason to believe with reasonable certainty that any taxon has disappeared from the region.

During the assessment process, the most frequently used criterion was B. Subcriteria B1 and B2 were mainly applied to species occurring in a small number of localities or in habitats whose extension or quality is declining. Criteria A, C and E were not used due to the lack of sufficient information on the size and dynamics of the bryophyte populations in Sicily, as well as in Italy [28]. The use of criterion D was minimal, being limited only to those cases in which a plausible threat could rapidly cause the extinction of the taxon or make it Critically Endangered.

Regarding the most endangered taxa, namely those classified as CR, EN and VU, they are mostly located in the main Sicilian mountain complexes, or occur on some smaller islands, and very rarely even in urban areas. They occupy a wide range of habitats. Some taxa (such as *Bazzania trilobata* var. *trilobata*, *Calypogeia muelleriana* subsp. *muelleriana*, *Riella macrocarpa* and *R. notarisii*) grow in humid environments, mostly represented by seasonal watercourses, mountain wetlands and brackish waters, others in forests (such as *Cololejeunea rossettiana* and *Targionia lorbeeriana*), in some cases also in planted woods (e.g., *Frullania parvistipula*), still others grow in scrub and garrigue formations (e.g., *Exormotheca pustulosa*), in grasslands (e.g., *Fossombronia caespitiformis* subsp. *multispira*), or even in urban areas (such as *Riccia atromarginata* var. *atromarginata* and *Sphaerocarpos europaeus*).

From a biogeographical perspective, some taxa exhibit a boreal distribution. These taxa are often at the southern limit of their range in Sicily and have found refuge at higher altitudes, where, in light of ongoing climate change, they may currently face population decline. This group includes *Barbilophozia barbata*, *B. hatcheri*, *Calypogeia muelleriana*, *Lophozia ventricosa*, *Mesoptychia collaris* and *Metzgeria pubescens*. Other species have a temperate distribution, such as *Cephaloziella rubella*, *Jungermannia atrovirens*, *J. pumila*, *Lophocolea minor* and *Nardia scalaris*, some of which are also at the southern limit of their range. Several taxa are Oceanic–Mediterranean, including *Cephaloziella stellulifera*, *Clevea spathysii*, *Exormotheca pustulosa*, *Fossombronia caespitiformis* subsp. *multispira*, *Microlejeunea ulicina*, *Riccia atromarginata*, *R. ciliifera*, *R. trabutiana* and *Targionia lorbeeriana*. These taxa often occur in coastal environments, in tourist–recreational sites, or in areas generally exposed to strong anthropogenic pressure.

A small group of eight taxa is considered at risk throughout the Italian territory [28]. These taxa, mostly Mediterranean or Oceanic–Mediterranean and very rare in Italy, include *Exormotheca pustulosa*, *Microlejeunea ulicina*, *Riccia trabutiana* (in Italy known only from Sicily), *Riella notarisii*, *Sphaerocarpos europaeus*, *Targionia lorbeeriana*, and *Riccia atromarginata* [1]. The last taxon, together with *Asterella africana*, *Cephaloziella dentata* and *Marchantia paleacea*, are considered at risk throughout the European continent [10].

Among taxa assigned to the Data Deficient category because of scarcity of information due to different taxonomic or distributional reasons, there are two, *Riccia ciliata* and *Fossombronia mittenii*, which have been classified as DD also in Italy and Europe. The first is a taxonomically uncertain taxon, the second is known in very few locations in Western Europe and could have been confused with *Fossombronia wondraczekii*, of which it could represent an anomalous form according to Paton [35].

Similarly, the paucity of data regarding the species *Radula lindenbergiana* and *Porella baueri* is believed to be probably due to misidentification, in the first case with *Radula complanata* and in the second with the parental species, *P. baueri*, being of hybrid origin.

A high level of distributional uncertainty prevented the attribution of a different category to *Riccia papillosa*, reported generically for Sicily, without a precise location [36] and *Marsupella boeckii* (Austin) Lindb. ex Kaal, whose presence on the island is based solely on a personal communication by M. Aleffi on a *datum* of R. Dull (*in litteris*).

Furthermore, there is insufficient information on the current distribution of *Blasia pusilla*, *Frullania fragilifolia*, *Marchantia quadrata* subsp. *quadrata*, *Nardia geoscyphus* var. *geoscyphus* and *Porella arboris-vitae* subsp. *arboris-vitae*. For these species, in fact, only data prior to 1968 are known relating to various sites in which not enough research has been recently conducted to confirm or exclude their presence there [1].

The species *Riccardia palmata* has been assigned to the Not Applicable category because it was considered vagrant. In fact, initially found in a greenhouse at the Botanical Garden of Palermo [37], the subpopulation has not been observed again in the ten years following the first report [38] and its presence is therefore to be considered occasional and ephemeral.

## 3. Discussion

The results of the survey show that approximately one third of the Sicilian liverworts and hornworts are threatened. A comparison between the data of this Red List and the Italian one highlights that the percentage incidences of the different threat categories are similar in the two lists. The percentage of threatened species (CR + EN + VU) in Sicily is slightly higher (32.5% vs. 28.4%). On the contrary, the percentage of NT is slightly lower compared to the Italian Red List (10.4% vs. 11.2%). A more marked difference is observed for Data Deficient taxa (15.2% vs. 10.4%); in most cases, this reflects the fact that the Sicilian bryophyte flora, having been studied more extensively than that of other Italian regions in past centuries, still includes several old records that require confirmation.

The high number of threatened species reflects the conservation status of the environment in Sicily, which unfortunately shows the effects of a long and often problematic relationship between humans and the island’s natural ecosystems, both in ancient and recent times, frequently disregarding existing protection regulations.

The results obtained indicate that, for the conservation of bryophytes, it is important to direct protection efforts not only towards mountain areas, where most of the protected sites are currently located, but also towards coastal areas, which are still poorly protected and are constantly exposed to strong anthropogenic impact. Furthermore, the results highlight the need to promote behaviours that are as respectful as possible of the environment as a whole, including habitats such as garrigues and grasslands, which are often not protected because they are considered to have a low degree of naturalness but which, as shown here, can host significant components of Sicilian and Italian biodiversity.

An analysis of the threat status of Sicilian liverworts also allows some considerations regarding the decline of specific habitats. The lack of respect for the environment has in fact led to the disappearance, or to a significant reduction, of many humid habitats of relevant conservation interest. These include, for example, very small *Sphagnum* bogs and acidic or basic marshes locally known as “margi”, which represent rare habitats in the Mediterranean area because they host numerous boreal species and provide evidence of the southward expansion of Arctic glaciers during the Quaternary. Similarly, the humid environments of retrodunal systems have suffered and, unfortunately, continue to suffer the effects of intense and often uncontrolled land sealing, aimed, for instance, at the construction of houses or tourist facilities even in close proximity to the coastline. This highlights the importance of actions focused on awareness raising and environmental education in order to preserve what still remains, especially in light of ongoing climate change, which is becoming increasingly severe and is expected to affect some regions of the Earth, including the Mediterranean Basin, particularly strongly. In this context, Red Lists can serve as useful tools to inform and raise awareness, contributing to the development of environmental consciousness.

In light of these considerations, this Red List assumes, in our opinion, a dual significance. On the one hand, it provides a valuable tool to support conservation actions for a group of plants that is often overlooked by non-specialists and is highly sensitive to environmental alteration. On the other hand, being the first Red List for the Sicilian bryophyte flora, it may represent a reference point for future monitoring of its conservation status. As such monitoring should ideally rely on quantitative data on population sizes and their temporal trends, we hope that similar studies can be carried out in the future, despite the many difficulties, primarily related to the limited number of young bryologists currently active in Sicily.

## 4. Materials and Methods

### 4.1. The Study Area

This study focuses on Sicily and its surrounding islets. The attention devoted to this area stems from two main factors. The first concerns the remarkable interest of the island. Indeed, Sicily is recognized as one of the major biodiversity hotspots in the Mediterranean area and it appears to be a very peculiar region due to its geographic position and morphology. It is, in fact, the largest island in the Mediterranean Basin and it is located approximately at its centre, representing a bridge between Africa and Italy as well as a connection between the western and eastern Mediterranean. The second element considered is the high vulnerability of the Mediterranean, and Sicily, to climate change, as stated by many authors [39,40,41].

Overall, Sicily covers a total area of 25,832.4 km^2^ [42]. In the northern part of the Island, the Madonie, Nebrodi, and Peloritani mountain ranges run roughly east–west and include the highest peaks, with Pizzo Carbonara in the Madonie reaching 1979 m. Mount Etna, located on the eastern coast, is the highest volcano in Europe, with a basal area of approximately 200,000 ha and an elevation exceeding 3300 m a.s.l. The remainder of the region consists of mountain complexes reaching lower altitudes, as well as hilly, flat or coastal areas. Specifically, lowlands (below 300 m a.s.l.) account for 14.20% of the total surface, while hills (300–700 m a.s.l.) represent 61.40% and mountainous areas (above 700 m a.s.l.) 24.50% [43].

Regarding geological features, sedimentary rocks are the most common on the island. Overall, the following types of lithological substrates are present: alluvial and coastal deposits; gessoso-solfifera formation in central Sicily; calcareous or calcareous–dolomitic formations, present mainly in the Madonie and in the mountains of Palermo and Trapani; metamorphic formations especially in the north-eastern part of Sicily in the Peloritani Mountains; arenaceous and argillaceous formations as in the Nebrodi Mountains; arenaceous or calcarenitic-sandy rocks as in the Erei Mountains; argillaceous formations, such as those surrounding many peaks in the Trapani area and also Mount Busambra and Mount Kumeta; volcanic and other magmatic rocks in the eastern part of Sicily, in the Etna area, in the Iblean area and in some of the islets [44,45].

The climate of Sicily is Mediterranean, with mild and rainy winters and hot and dry summers, but varies according to altitude and slope. The typical Mediterranean climatic characteristics are generally found in the coastal strips and coastal plains, while towards the mountainous inland the thermal and rainfall conditions undergo variations due to altitude and the progressively weaker influence of the sea.

The average annual temperature is quite high everywhere, ranging from 19 °C in the coastal areas to around 13 °C in the highest areas of inland. The annual temperature variation is moderate along the coasts especially the southern and western ones, while it exceeds 20 °C in some inland areas.

The predominant winds affecting Sicily are the Mistral and Tramontana, particularly during winter, the Libeccio mainly in spring and autumn and the Sirocco, which, especially in summer, is the cause of large heat waves with temperatures that sometimes exceed 40 °C and transport of dust from the desert. The northern winds are instead the cause of intense rains on the northern and eastern sides of the island. Precipitation is not abundant, with an average of 500–700 mm/year. It shows significant interannual variability and is unevenly distributed both throughout the year and across the island’s different geographic sectors. About 70–80% of the rain falls between October and March, with maximum rainfall in November. Precipitation often takes on a violent character with dangerous consequences especially on the clayey soils of the Sicilian inland. The rainiest areas coincide with the main mountain ranges of the island, where on average between 600 and 700 and 1400 and 1600 mm of rain fall per year, with peaks of 1800–2000 mm at the highest altitudes of Mount Etna. In contrast, the driest areas of the island, where rainfall can drop below 300 mm, are the south-eastern ones (Piana di Catania, Piana di Gela, part of the province of Enna), as well as the extreme western and southern areas. In the remaining parts of Sicily, average rainfall generally ranges from about 300 to 400 to 700 to 800 mm per year [46,47].

According to the bioclimatic classification of Rivas-Martínez et al. [48], the bioclimate is Mediterranean pluviseasonal oceanic, ranging from the lower thermo-Mediterranean to the lower meso-Mediterranean thermotype and from the lower dry to the upper semiarid ombrotype [49].

### 4.2. Data Sources

The list of taxa and their distributional data were derived from the Sicilian bryophyte database curated at the Bryology Laboratory of the Department of Biological, Chemical and Pharmaceutical Sciences and Technologies, University of Palermo. In addition to records collected over the last 50 years, the database incorporates historical reports dating back to 1806, as compiled in the only comprehensive checklist of Sicilian liverworts currently available [50]. In addition to published reports, herbarium specimens (CAT and PAL) and the results of recent field surveys conducted by the authors were considered.

The species and infraspecific taxa listed correspond to those reported for Sicily in the checklist of Italian bryophytes [1], with the addition of the species *Asterella africana* and *Riella macrocarpa*, recently, recorded in Sicily [51,52]. In accordance with this checklist, all records dating from 1968 onwards were considered recent and were used for the calculation of Extent of Occurrence (EOO) and Area of Occupancy (AOO). Records prior to this date were instead taken into account for the assessment of population decline.

The nomenclature and taxonomy of taxa follow Hodgetts et al. [53]. Chorological categories were assigned according to Hill et al. [54]; for taxa not covered by that contribution, the classification proposed by Düll [55] was adopted. The conservation status of each taxon in Italy and Europe was derived from the Italian [28] and the European Red List of bryophytes [10], respectively.

### 4.3. Assessment Methods

The conservation status of the selected taxa was assessed according to the criteria and categories of IUCN [30,31,34], and considering the guidelines for bryophytes [56,57,58].

According to the IUCN [32], nine categories were used: RE (Regional Extinct), CR (PE) (Critically Endangered Possibly Extinct), CR (Critically Endangered), EN (Endangered), VU (Vulnerable), NT (Near Threatened), DD (Data Deficient), LC (Least Concern), and NA (Not Applicable). In particular, the Near Threatened (NT) category was applied to taxa close to qualifying for the Vulnerable (VU) category, and in all cases the criterion that was nearly met was indicated. In particular, these taxa usually met the area requirements under criterion B for threatened EOO and/or AOO without fulfilling at least two of the conditions A, B and C. The Data Deficient (DD) category was applied to taxa for which current information on distribution, population size, trends, ecology and threats is insufficient to assess their level of risk, making the status of the species so uncertain that both Critically Endangered (CR) and Least Concern (LC) can represent plausible categories [44].

The assessments were mostly based on criterion B, since in Sicily, as well as in Italy and other European Countries, data on the bryophyte population trend, useful for the application of criteria A and C, are very scarce. In particular, we decide that continuing decline of the population is assessed, and therefore Criterion B is applied instead of the Criterion D, when the threat to the species persists over time.

Distribution data were mainly drawn from published and unpublished data, herbarium specimens, and recent field surveys of the Authors.

For all assessments, the following information was collected: updated taxonomic status of taxa; georeferenced distribution records; habitat and ecological requirements; threat category in neighbouring countries and in Europe; actual and potential threats; incidence of old reports not recently confirmed; conservation measures. Distribution data were used for calculating EOO and AOO, obtained through GeoCAT (Geospatial Conservation Assessment Tool), (https://geocat.iucnredlist.org/), (accessed on 10 September 2025, Palermo, Italy) an open-source browser based tool developed by Kew Gardens to utilize spatially referenced primary occurrence data, performing rapid geospatial analysis to ease the process of Red Listing taxa. The grid for the calculation of the Area of Occupancy was 4 km^2^ (2 × 2 km). Data on habitat and ecology were drawn from BRYOATT [54], the risk status of taxa in Italy [28] and Europe [10]. The conservation measures considered are indirect (species within natural reserves or parks) in absence of action plans for the protection of individual species in Sicily.

In applying the IUCN criteria, 1968 has been chosen as the cut-off date to represent the threshold between old and recent records for the purpose of assessing decline [1,28,29].

The presence or absence of current or potential future threats to population persistence in Sicilian localities was assessed on the basis of the authors’ personal knowledge of the sites and general information on threats to taxa and their habitats known in Europe [10]. At the same time, any form of protection or management affecting the sites that could support population survival was taken into account, including Nature Reserves and habitats of community interest identified under the Habitats Directive 92/43/EEC.

Furthermore, during the evaluation of taxa, and in the absence of specific studies, factors potentially affecting the possibility of recolonization from other territories were considered. These evaluation elements mainly concerned the distribution of species in neighbouring areas (the Italian peninsula or the Mediterranean region) and the dispersal capacity of bryophytes, which depends on different biological and ecological traits, as highlighted by numerous authors, e.g., [59,60].

Given the lack of precise knowledge regarding both the Sicilian and overall distribution that characterizes bryophytes as a taxonomic group, a precautionary but realistic approach to uncertainty was adopted, following the recommendations of the IUCN [34].

In Table 1, in addition to the assessed taxa (listed in alphabetical order by genus), the families, the threat categories in Sicily, the applied criteria, and the threat categories at the Italian and European levels are reported.

## Figures and Tables

**Table 1:** List of Sicilian liverworts and hornworts, with corresponding IUCN Red List categories and assessment criteria.

Taxon	Family	Threat Category	Criteria	Italian Threat Category	European Threat Category
*Aneura pinguis* (L.) Dumort.	*Aneuraceae*	LC		LC	LC
*Anthoceros agrestis* Paton	*Anthocerotaceae*	NT	B1a, B2a	NT	NT
*Anthoceros punctatus* L.	*Anthocerotaceae*	LC		LC	LC
*Apopellia endiviifolia* (Dicks.) Nebel & D. Quandt	*Pelliaceae*	LC		LC	LC
*Asterella africana* (Mont.) Underw. ex A. Evans	*Aytoniaceae*	CR	B2ab (iii)	DD	VU
*Barbilophozia barbata* (Schmidel ex Schreb.) Loeske	*Anastrophyllaceae*	CR	B2ab (iii)	LC	LC
*Barbilophozia hatcheri* (A.Evans) Loeske	*Anastrophyllaceae*	CR	B2ab (iii)	LC	LC
*Bazzania trilobata* (L.) Gray var. *trilobata*	*Lepidoziaceae*	EN	B1ab (iii), B2ab (iii)	LC	LC
*Blasia pusilla* L.	*Blasiaceae*	DD		LC	LC
*Calypogeia arguta* Nees & Mont.	*Calypogeiaceae*	LC		LC	LC
*Calypogeia azurea* Stotler & Crotz	*Calypogeiaceae*	NT	B1a, B2a	LC	LC
*Calypogeia fissa* (L.) Raddi	*Calypogeiaceae*	LC		LC	LC
*Calypogeia muelleriana* (Schiffn.) Müll.Frib. subsp. *muelleriana*	*Calypogeiaceae*	CR	B2ab (iii)	LC	LC
*Calypogeia sphagnicola* (Arnell & J. Perss.) Warnst. & Loeske	*Calypogeiaceae*	DD		EN	LC
*Cephalozia bicuspidata* (L.) Dumort subsp. *bicuspidata*	*Cephaloziaceae*	LC		LC	LC
*Cephaloziella baumgartneri* Schiffn.	*Cephaloziellaceae*	LC		LC	LC
*Cephaloziella calyculata* (Durieu & Mont.) Müll.Frib.	*Cephaloziellaceae*	EN	B2ab (ii, iv)	NT	NT
*Cephaloziella dentata* (Raddi) Steph.	*Cephaloziellaceae*	EN	B1ab (ii, iv), B2ab (ii, iv)	NT	EN
*Cephaloziella divaricata* (Sm.) Schiffn. var. *divaricata*	*Cephaloziellaceae*	LC		LC	LC
*Cephaloziella rubella* (Nees) Warnst.	*Cephaloziellaceae*	VU	B2ab (iii)	LC	LC
*Cephaloziella stellulifera* (Taylor ex Carrington & Pearson) Croz.	*Cephaloziellaceae*	VU	D2	LC	LC
*Cephaloziella turneri* (Hook.) Müll.Frib.	*Cephaloziellaceae*	NT	B1b (ii, iv), B2b (ii, iv)	LC	LC
*Chiloscyphus pallescens* (Ehrh.) Dumort. var. *pallescens*	*Lophocoleaceae*	EN	B1ab (iii), B2ab (iii)	LC	LC
*Chiloscyphus polyanthos* (L.) Corda var. *polyanthos*	*Lophocoleaceae*	EN	B1ab (iii), B2ab (iii)	LC	LC
*Clevea spathysii* (Lindenb.) Müll.Frib.	*Cleveaceae*	VU	B2ab (iii)	DD	NT
*Cololejeunea rossettiana* (C. Massal.) Schiffn.	*Lejeuneaceae*	CR	B2ab (iii)	LC	LC
*Conocephalum conicum* (L.) Dumort.	*Conocephalaceae*	LC		LC	LC
*Corsinia coriandrina* (Spreng.) Lindb.	*Corsiniaceae*	LC		LC	LC
*Diplophyllum albicans* (L.) Dumort.	*Scapaniaceae*	VU	B1ab (ii, iii, iv), B2ab (ii, iii, iv)	LC	LC
*Exormotheca pustulosa* Mitt.	*Exormothecaceae*	EN	B1ab (iii), B2ab (iii)	CR	NT
*Fossombronia angulosa* (Dicks.) Raddi	*Fossombroniaceae*	LC		LC	LC
*Fossombronia caespitiformis* (Raddi) De Not. ex Rabenh. subsp. *caespitiformis*	*Fossombroniaceae*	LC		LC	LC
*Fossombronia caespitiformis* (Raddi) De Not. ex Rabenh. subsp. *multispira* (Schiffn.) J.R. Bray & Cargill	*Fossombroniaceae*	VU	B1ab (ii, iii, iv), B2ab (ii, iii, iv)	NT	LC
*Fossombronia echinata* Macvicar	*Fossombroniaceae*	CR	B2ab (iii)	NT	NT
*Fossombronia mittenii* Tind.	*Fossombroniaceae*	DD		DD	DD
*Fossombronia pusilla* (L.) Nees	*Fossombroniaceae*	LC		LC	LC
*Fossombronia wondraczekii* (Corda) Dumort. ex Lindb.	*Fossombroniaceae*	LC		NT	LC
*Frullania dilatata* (L.) Dumort. subsp. *dilatata*	*Frullaniaceae*	LC		LC	LC
*Frullania fragilifolia* (Taylor) Gottsche, Lindenb. & Nees	*Frullaniaceae*	DD		LC	LC
*Frullania parvistipula* Steph.	*Frullaniaceae*	DD		EN	CR
*Frullania tamarisci* (L.) Dumort.	*Frullaniaceae*	LC		LC	LC
*Gongylanthus ericetorum* (Raddi) Nees	*Southbyaceae*	LC		LC	LC
*Jungermannia atrovirens* Dumort.	*Jungermanniaceae*	EN	B1ab (ii, iii, iv), B2ab (ii, iii, iv)	LC	LC
*Jungermannia eucordifolia* Schljakov	*Jungermanniaceae*	DD		LC	LC
*Jungermannia pumila* With.	*Jungermanniaceae*	DD		LC	LC
*Lejeunea cavifolia* (Ehrh.) Lindb.	*Lejeuneaceae*	LC		LC	LC
*Lophocolea bidentata* (L.) Dumort.	*Lophocoleaceae*	LC		LC	LC
*Lophocolea heterophylla* (Schrad.) Dumort. subsp. *heterophylla*	*Lophocoleaceae*	LC		LC	LC
*Lophocolea minor* Nees	*Lophocoleaceae*	VU	D2	LC	LC
*Lophozia ventricosa* (Dicks.) Dumort.	*Lophoziaceae*	CR	B2ab (iii)	LC	LC
*Lophoziopsis excisa* (Dicks.) Konstant. & Vilnet var. *excisa*	*Lophoziaceae*	CR	B2ab (iii)	LC	LC
*Lunularia cruciata* (L.) Dumort. ex Lindb. subsp. *cruciata*	*Lunulariaceae*	LC		LC	LC
*Mannia androgyna* (L.) A. Evans	*Aytoniaceae*	LC		LC	LC
*Mannia fragrans* (Balb.) Frye & L.Clark subsp. *fragrans*	*Aytoniaceae*	DD		VU	VU
*Marchantia paleacea* Bertol. Subsp. *paleacea*	*Marchantiaceae*	VU	B1ab (ii, iii, iv), B2ab (ii, iii, iv)	LC	VU
*Marchantia polymorpha* L. subsp. *ruderalis* Bischl. & Boissel. -Dub.	*Marchantiaceae*	LC		LC	LC
*Marchantia quadrata* Scop. subsp. quadrata	*Marchantiaceae*	DD		LC	LC
*Marsupella boeckii* (Austin) Lindb. ex Kaal.	*Gymnomitriaceae*	DD		DD	LC
*Marsupella emarginata* (Ehrh.) Dumort.	*Gymnomitriaceae*	LC		LC	LC
*Mesoptychia collaris* (Nees) L. Söderstr. & Váňa	*Jungermanniaceae*	CR	B2ab (iii)	LC	LC
*Mesoptychia turbinata* (Raddi) L. Söderstr. & Váňa	*Jungermanniaceae*	LC		LC	LC
*Metzgeria furcata* (L.) Corda	*Metzgeriaceae*	LC		LC	LC
*Metzgeria pubescens* (Schrank) Raddi	*Metzgeriaceae*	CR	B2ab (iii)	LC	LC
*Microlejeunea ulicina* (Taylor) Steph.	*Lejeuneaceae*	VU	D2	VU	LC
*Myriocoleopsis minutissima* (Sm.) R.L. Zhu, Y.Yu & Pócs subsp. *minutissima*	*Lejeuneaceae*	NT	B1a, B2a	LC	LC
*Nardia geoscyphus* (De Not.) Lindb. var. *geoscyphus*	*Gymnomitriaceae*	DD		LC	LC
*Nardia scalaris* Gray var. *scalaris*	*Gymnomitriaceae*	EN	B1ab (ii, iv), B2ab (ii, iv)	LC	LC
*Neoorthocaulis floerkei* (F. Weber & D.Mohr) L.Söderstr., De Roo & Hedd.	*Anastrophyllaceae*	EN	B1ab (iii), B2ab (iii)	NT	LC
*Oxymitra incrassata* (Brot.) Sérgio & Sim-Sim	*Oxymitraceae*	LC		LC	LC
*Pedinophyllum interruptum* (Nees) Kaal.	*Plagiochilaceae*	NT	B2a	LC	LC
*Pellia epiphylla* (L.) Corda subsp. *epiphylla*	*Pelliaceae*	LC		LC	LC
*Petalophyllum ralfsii* (Wilson) Nees & Gottsche	*Petalophyllaceae*	LC		NT	LC
*Phaeoceros laevis* (L.) Prosk.	*Notothyladaceae*	LC		LC	LC
*Phymatoceros bulbiculosus* (Brot.) Stotler, W.T.Doyle & Crand.-Stotl.	*Phymatocerotaceae*	LC		LC	LC
*Plagiochasma rupestre* (J.R.Forst. & G.Forst.) Steph. var. rupestre	*Aytoniaceae*	LC		LC	LC
*Plagiochila asplenioides* (L.) Dumort.	*Plagiochilaceae*	LC		LC	LC
*Plagiochila porelloides* (Torr. ex Nees) Lindenb. var. *porelloides*	*Plagiochilaceae*	LC		LC	LC
*Porella arboris-vitae* (With.) Grolle subsp. *arboris-vitae*	*Porellaceae*	DD		LC	NT
*Porella baueri* (Schiffn.) C.E.O. Jensen	*Porellaceae*	DD		DD	DD
*Porella cordaeana* (Huebener) Moore	*Porellaceae*	LC		LC	LC
*Porella obtusata* (Taylor) Trevis.	*Porellaceae*	NT	B1a, B2a	LC	LC
*Porella platyphylla* (L.) Pfeiff.	*Porellaceae*	LC		LC	LC
*Radula complanata* (L.) Dumort.	*Radulaceae*	LC		LC	LC
*Radula lindenbergiana* Gottsche ex C. Hartm.	*Radulaceae*	DD		LC	LC
*Reboulia hemisphaerica* (L.) Raddi subsp. *hemisphaerica*	*Aytoniaceae*	LC		LC	LC
*Riccardia chamedryfolia* (With.) Grolle	*Aneuraceae*	NT	B1a, B2a	LC	LC
*Riccardia multifida* (L.) Gray subsp. *multifida*	*Aneuraceae*	LC		LC	LC
*Riccardia palmata* (Hedw.) Carruth.	*Aneuraceae*	NA		LC	LC
*Riccia atromarginata* Levier var. *atromarginata*	*Ricciaceae*	VU	B1ab (ii, iii, iv), B2ab (ii, iii, iv)	EN	EN
*Riccia bicarinata* Lindb.	*Ricciaceae*	NT	B1a, B2a	VU	LC
*Riccia bifurca* Hoffm.	*Ricciaceae*	DD		DD	LC
*Riccia cavernosa* Hoffm.	*Ricciaceae*	CR	B2ab (iii)	LC	LC
*Riccia ciliata* Hoffm.	*Ricciaceae*	DD		DD	DD
*Riccia ciliifera* Link	*Ricciaceae*	CR	B2ab (iii)	LC	LC
*Riccia crozalsii* Levier	*Ricciaceae*	CR	B2ab (iii)	LC	LC
*Riccia crystallina* L.	*Ricciaceae*	LC		LC	LC
*Riccia fluitans* L.	*Ricciaceae*	CR	B2ab (iii)	LC	LC
*Riccia glauca* L. var. *glauca*	*Ricciaceae*	LC		LC	LC
*Riccia gougetiana* Durieu & Mont. var. *gougetiana*	*Ricciaceae*	NT	B1a, B2a	LC	LC
*Riccia lamellosa* Raddi	*Ricciaceae*	LC		LC	LC
*Riccia macrocarpa* Levier	*Ricciaceae*	NT	B1a, B2a	NT	LC
*Riccia michelii* Raddi	*Ricciaceae*	NT	B2a	LC	LC
*Riccia nigrella* DC.	*Ricciaceae*	LC		LC	LC
*Riccia papillosa* Moris	*Ricciaceae*	DD		DD	LC
*Riccia sorocarpa* Bisch. subsp. *sorocarpa*	*Ricciaceae*	LC		LC	LC
*Riccia trabutiana* Steph.	*Ricciaceae*	EN	B2ab (ii, iii, iv)	CR	LC
*Riccia warnstorfii* Limpr. ex Warnst.	*Ricciaceae*	LC		LC	VU
*Riella macrocarpa* (P. Allorge) Puche, Segarra, Sabovlj., M. Infante & Heras	*Riellaceae*	CR	B2ab (iii)	CR	-
*Riella notarisii* (Mont.) Mont.	*Riellaceae*	CR	B2ab (iii)	EN	NT
*Scapania aequiloba* (Schwägr.) Dumort.	*Scapaniaceae*	CR	B2ab (iii)	LC	LC
*Scapania aspera* M. Bernet & Bernet	*Scapaniaceae*	NT	B1a, B2a	LC	LC
*Scapania compacta* (Roth) Dumort.	*Scapaniaceae*	LC		LC	LC
*Scapania curta* (Mart.) Dumort. var. *curta*	*Scapaniaceae*	DD		NT	LC
*Scapania nemorea* (L.) Grolle	*Scapaniaceae*	DD		LC	LC
*Scapania subalpina* (Nees ex Lindenb.) Dumort. var. *subalpina*	*Scapaniaceae*	DD		LC	LC
*Scapania undulata* (L.) Dumort.	*Scapaniaceae*	LC		LC	LC
*Solenostoma gracillimum* (Sm.) R.M.Schust.	*Solenostomataceae*	LC		LC	LC
*Solenostoma hyalinum* (Lyell) Mitt.	*Solenostomataceae*	NT	B1a, B2a	LC	LC
*Solenostoma obovatum* (Nees) C. Massal.	*Solenostomataceae*	EN	B1ab (ii, iii, iv), B2ab (ii, iii, iv)	LC	LC
*Solenostoma sphaerocarpum* (Hook.) Steph.	*Solenostomataceae*	EN	B1ab (iii), B2ab (iii)	LC	LC
*Southbya nigrella* (De Not.) Henriq.	*Southbyaceae*	LC		LC	LC
*Southbya tophacea* (Spruce) Spruce	*Southbyaceae*	LC		LC	LC
*Sphaerocarpos europaeus* Lorb.	*Sphaerocarpaceae*	CR	B2ab(iii)	EN	LC
*Sphaerocarpos michelii* Bellardi	*Sphaerocarpaceae*	LC		LC	LC
*Targionia hypophylla* L. subsp. *hypophylla*	*Targioniaceae*	LC		LC	LC
*Targionia lorbeeriana* Müll.Frib.	*Targioniaceae*	EN	B1ab (ii, iii, iv), B2ab (ii, iii, iv)	EN	LC

## Data Availability

Data are contained within the article.
